# Genetic and Epigenetic Modifiers of Alcoholic Liver Disease

**DOI:** 10.3390/ijms19123857

**Published:** 2018-12-03

**Authors:** Marica Meroni, Miriam Longo, Raffaela Rametta, Paola Dongiovanni

**Affiliations:** General Medicine and Metabolic Diseases, Fondazione IRCCS Ca’ Granda Ospedale Maggiore Policlinico, Pad. Granelli, via F Sforza 35, 20122 Milan, Italy; maricameroni11@gmail.com (M.M.); longo.miriam92@gmail.com (M.L.); raffaela.rametta@policlinico.mi.it (R.R.)

**Keywords:** alcoholic liver disease, PNPLA3, TM6SF2, MBOAT7, microRNAs, epigenetics, intestinal permeability, tight junctions

## Abstract

Alcoholic liver disease (ALD), a disorder caused by excessive alcohol consumption is a global health issue. More than two billion people consume alcohol in the world and about 75 million are classified as having alcohol disorders. ALD embraces a wide spectrum of hepatic lesions including steatosis, alcoholic steatohepatitis (ASH), fibrosis, cirrhosis, and hepatocellular carcinoma (HCC). ALD is a complex disease where environmental, genetic, and epigenetic factors contribute to its pathogenesis and progression. The severity of alcohol-induced liver disease depends on the amount, method of usage and duration of alcohol consumption as well as on age, gender, presence of obesity, and genetic susceptibility. Genome-wide association studies and candidate gene studies have identified genetic modifiers of ALD that can be exploited as non-invasive biomarkers, but which do not completely explain the phenotypic variability. Indeed, ALD development and progression is also modulated by epigenetic factors. The premise of this review is to discuss the role of genetic variants and epigenetic modifications, with particular attention being paid to microRNAs, as pathogenic markers, risk predictors, and therapeutic targets in ALD.

## 1. Introduction

Chronic alcohol consumption is one of the leading causes of mortality worldwide [1]. According to the World Health Organization (WHO), 2.3 billion people consume alcohol in the world and about 75 million are classified as having alcohol disorders [2]. In 2012, approximately 5.9% of global deaths were attributed to alcohol abuse [1,3]. The liver is the primary site of ethanol metabolism, thus sustaining the greatest degree of tissue injury by heavy drinking. Alcoholic liver disease (ALD) embraces a broad spectrum of hepatic lesions including steatosis, alcoholic steatohepatitis (ASH), fibrosis, cirrhosis, and hepatocellular carcinoma (HCC) [4]. The histopathological features of ALD are similar to those observed in nonalcoholic fatty liver disease (NAFLD), which is an alcohol-like liver disease in absence of alcohol abuse (<20 g/day for women and <30 g/day for men), mainly attributable to obesity and the metabolic syndrome (MetS) [5,6]. The histological similarities make it harder to distinguish between ALD and NAFLD [7]. However, several hallmarks may be suggestive of alcoholic etiology rather than non-alcoholic (i.e., high density of Mallory–Denk bodies, central hyaline sclerosis, lipogranulomas, and cholestasis) [8]. Moreover, differences in inflammatory cell infiltration, characterized by a higher rate of neutrophils surrounding ballooned hepatocytes (‘satellitosis’) and portal inflammation and necrosis are more common in ALD than NAFLD. 

Many other aspects contribute to differentiate these two chronic liver diseases, ranging from epidemiology to clinical features and patient outcomes [7,9].

The correlation between alcohol consumption and liver disease is now widely recognized and the majority of individuals (90%) who regularly consume more than 40–60 g/day of alcohol develop steatosis. However, steatosis also develops after binge drinking, defined as the consumption of four of five drinks in two hours or less. If the affected individual ceases drinking, steatosis is a reversible condition. In 20–40% of dependent drinkers, the presence of steatosis may be complicated by the development of inflammation and fibrosis while cirrhosis develops in about 10–20% [10]. 

HCC annually occurs in 2–3% of both alcoholic and nonalcoholic-related cirrhosis [11,12,13] and only half of the patients are eligible for therapeutic treatments (liver transplantation, resection, and percutaneous ablation) [13]. 

ALD and NAFLD are considered complex ‘multifactorial diseases’, in which environmental, genetic, and epigenetic factors cause differences in susceptibility to liver damage, even influencing the pathological phenotype and progression. 

## 2. Pathogenesis, Natural History, and Clinical Aspects of Alcoholic Liver Disease (ALD)

The liver and to a lesser extent the gastrointestinal tract are mainly involved in alcohol metabolism. Ethanol is firstly oxidized to acetaldehyde by Alcohol Dehydrogenase (ADH), a cytosolic enzyme which uses NAD^+^ as cofactor. Acetaldehyde is highly toxic, and it can bind proteins, DNA or lipids by forming adducts with these macromolecules thus impairing their function. To limit its reactivity, acetaldehyde is further oxidized to acetate by Aldehyde Dehydrogenase (ALDH) which is localized in the mitochondria and similarly to ADH uses NAD^+^ as cofactor. ADH and ALDH reactions lead to a reduced NAD^+^/NADH ratio which impacts oxidative metabolism by favoring fatty acid synthesis and fat accumulation [14]. NADH is re-oxidized to NAD^+^ in the mitochondria thus generating reactive oxygen species (ROS) which can affect lipid peroxidation and DNA mutagenesis. 

Another enzyme involved in ethanol metabolism is the Cytochrome P450 2E1(CYP2E1) which is induced by chronic alcohol consumption [15] and catalyzes ethanol oxidation to acetaldehyde by generating a significant amount of ROS, triggers of oxidative stress and inflammation. 

Acetaldehyde, as well as, CYP2E1-derived oxidative stress inhibits Peroxisome Proliferator Activated Receptor α (PPARα) transcriptional activity and decreases PPARα targets genes [16] thus leading to reduced fatty acid oxidation. Similarly, acetaldehyde enhances sterol regulatory element-binding protein (Srebp-1c) activity by favoring its translocation from endoplasmic reticulum (ER) to Golgi apparatus where it undergoes proteolytic maturation to its active form and promotes de novo lipogenesis. In addition, animal studies revealed that chronic alcohol consumption decreases adipose tissue mass by favoring lipolysis and free fatty acids (FFAs) flux to the liver where they are esterified in triglycerides [17]. Clinical data confirmed that ALD patients with fatty liver have lower body mass index (BMI) and body weight compared to control subjects [18]. Overall, de novo lipogenesis induction—the generation of lipid peroxidation products along with ethanol-induced deranged coagulation—may lead even to extra-hepatic complications, increasing cardiovascular risk in alcoholics [19]. 

Acetaldehyde seems to be involved not only in steatosis development which occurs in 90% of alcohol abusers but also in alcohol-related fibrogenesis by enhancing synthesis of collagen and transforming growth factor beta (TGFβ) secretion in hepatic stellate cells (HSCs) [20,21]. Activated HSCs also contribute to the inflammatory response by releasing cytokines and chemokines and favoring the recruitment of inflammatory cells which leads to steatohepatitis development. Alcoholic steatohepatitis and hepatic fibrosis are reversible condition if alcohol consumption ceases. Otherwise, the chronicization of inflammation and fibrogenesis may progress into liver cirrhosis. Unlike steatosis which is associated with alcohol consumption but rarely progresses to cirrhosis, alcoholic steatohepatitis is a rate limiting step for the development of cirrhosis and almost 40% of patients with ASH progress to cirrhosis within five years [22]. In a Danish National Registry study, patients with steatohepatitis are more susceptible to develop cirrhosis compared to patients affected by simple steatosis (16% vs. 6.9%) [23]. Cirrhosis is a risk factor of HCC development and different mechanism may contribute to HCC onset in ALD. Acetaldehyde has mutagenic properties whereas CYP2E1 metabolizes pro-carcinogenic compounds which are present in alcoholic drinks. Finally, higher levels of lipopolysaccharides (LPS) in ALD patients promote cancer stem cells proliferation [24].

## 3. Environmental Risk Factors Involved in ALD Pathogenesis

Several factors have been identified at individual and society levels to affect the pattern or extent of alcohol consumption and the risk to develop liver diseases. 

### 3.1. Alcohol Consumption 

The main epidemiological risk factor for ALD is excessive alcohol consumption. There is a great regional variability in alcohol consumption due to socioeconomic, cultural and religious factors with the highest levels in Europe and USA and the lowest ones in South-East Asia and eastern Mediterranean countries. Thus, alcohol-related diseases occur more frequently in the developed world [25]. Several large prospective cohort studies have shown that in individuals with high alcohol consumption (40 g/day for women and 60 g/day for men) there is a dose-dependent increase in cirrhosis risk [26,27,28]. A population study demonstrated that subjects with a daily intake greater than 30 g of alcohol had an enhanced risk of developing alcoholic liver injury and cirrhosis. Subjects who consumed more than 120 g/day of alcohol have increased risk of cirrhosis [29] although the length of time during which an individual has regularly drunk impacts the risk of cirrhosis more than the amount of alcohol consumed. Moreover, it has been described that daily drinking compared to episodic or binge drinking is associated with higher risk of cirrhosis [27]. In addition, consumption of alcohol with food is less harmful than drinking on an empty stomach and wine has less serious consequences on ALD development than beer, although this could be explained by the fact that beer drinkers consume more caloric diets [30,31]. 

### 3.2. Age 

Adolescents and elderly people are more vulnerable to alcohol-related diseases than other age groups [32,33]. Alcohol use before the age of 14 years as well as parental alcohol problems correlated with a high risk to develop alcohol abuse and dependence [34]. Older people consume alcohol more frequently than other age groups, but they are less able to metabolize it and more susceptible to be affected by alcohol-related problems [33]. 

### 3.3. Gender 

Gender also plays an important role in alcohol susceptibility. Men consume significantly more alcohol than women and consequently have nine times more alcohol-related liver disease [29]. However, for a given levels of drinking women are more vulnerable to alcohol-related liver damage than men. This appears to be related to higher blood alcohol concentrations in women than in men with the same amount of alcohol ingested [35] and to the effects of estrogen on alcohol-related liver injury. It has been described in rodents that chronic alcohol consumption makes macrophages more susceptible to endotoxin-mediated Tumor Necrosis Factor-alpha (TNFα) production and estrogens sensitize Kupffer cells to endotoxin injury [36]. Moreover, chronic alcohol abuse induces a pro-inflammatory response in adipose tissue possibly in a sex dependent manner with a greater inflammation that influence liver damage in female mice exposed to chronic-binge ethanol [37]. Several studies demonstrated that even NAFLD is more prevalent in males than in women. Conversely, in NAFLD patients, estrogens seem to have a protective effect. Indeed, estrogen deficiency and ovarian senescence due to old age may increase the susceptibility to NAFLD [38].

### 3.4. Ethnicity

There is a huge inter-ethnic variability in the predisposition towards ALD. Indeed, ethnicity represents one of the major factors which affect the development and outcome of ALD [39] and there are several inter-ethnic differences in alcohol-related cirrhosis risk. White Hispanic men, especially those of Mexican origin, have the highest risk of alcohol-related cirrhosis [40] and seem to develop ALD at a younger age than Caucasians and African Americans [39]. Notably, White Hispanics also display the highest incidence of NAFLD compared to other ethnicities [41]. Finally, American Indians and native Alaskans have a significantly greater mortality from chronic ALD compared to White people [42].

### 3.5. Obesity

Among environmental risk factors, obesity is the widest recognized. It has been described that overweight or obese women in the UK who consume low to moderate amounts of alcohol had growing risk of liver cirrhosis compared to women with a BMI between 22.5 and 25. The increase in liver cirrhosis rates with higher BMI was substantially greater in women who drank 150 g or more of alcohol than in those reporting drinking less than 70 g per week [43,44,45]. In a prospective cohort study with more than 9000 participants, Hart and colleagues investigated the combined effects of BMI and alcohol consumption on liver disease. Both factors were related to liver disease and there was a supra-additive interaction between the two. Obesity leads to steatohepatitis affecting hepatic insulin sensitivity and the lipid solubility of ethanol influences adipose tissue production of hormones and cytokines. Meanwhile, alcoholic fatty liver induces peripheral insulin resistance, thus promoting obesity [46,47]. 

It has been demonstrated that in patients with alcoholic liver disease, a previous history of excess weight was an independent risk factor for the pathogenesis of steatosis, fibrosis and cirrhosis [44,45,48]. Bellentani and colleagues found that alcohol and obesity are both associated with hepatic steatosis, whose prevalence is higher (94.5%) in obese drinkers [49].

Differently from NAFLD, high blood pressure was the only MetS component associated with steatosis in alcoholic patients [44]. However, Raynard et al. found that BMI, iron accumulation and blood glucose levels were independent risk factors for fibrosis in alcohol liver disease after adjustment for daily alcohol intake and duration of alcohol consumption [45].

## 4. Genetic Factors Involved in ALD Pathogenesis

Alcoholism has been considered to be a familial disorder. People with a familial history of alcohol abuse are more susceptible to develop alcohol dependence (49.7% for men and 22.4% for women) [50]. 

Twin, family, and adoption studies provide evidence for a significant hereditability of alcohol dependence, but the specific genetic risk factors involved in ALD pathogenesis remained to be elucidated. Walters et al. performed a meta-analysis which included 50 family, twin, and adoption studies. He found that the genetic impact on alcohol misuse was very heterogeneous between the studies evaluated and indicated an upper limit of 30–36% for the hereditability in men with severe alcohol abuse [51]. Verhulst et al. performed a meta-analysis which included 12 twin and 5 adoption studies and concluded that alcohol use disorder (AUD) is 50% hereditable and that environmental factors may contribute to the familial aggregation of AUDs [52]. Hrubec et al. estimated the hereditability for alcohol-related cirrhosis by evaluating 15,924 male twin-pairs in the National Academy of Sciences-National Research Council Twin Registry. The hereditability for alcohol-related cirrhosis ranged from 21% to 67% and was three times higher in monozygotic than in dizygotic twins [53].

**Figure 1:**
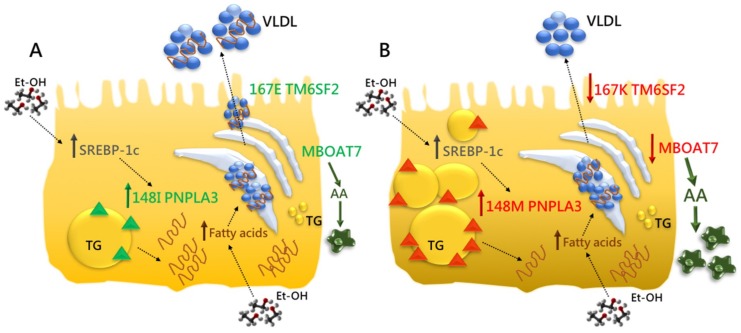
*PNPLA3*, *TM6SF2*, and *MBOAT7*: the genetic modifiers of ALD. (**A**) The *Patatin-like phospholipase domain-containing 3* (*PNPLA3*) is an intracellular membrane lipase, localized on the surface of lipid droplets in hepatocytes where catalyzed the hydrolysis of triglycerides (TG). The *Transmembrane 6 superfamily member 2* (*TM6SF2*) is involved in very low-density lipoprotein (VLDL) secretion, whereas the *Membrane bound O-acyltransferase domain containing 7* (*MBOAT7*) catalyzes the transfers of polyunsaturated fatty acids, such as arachidonoyl-CoA to lysophospholipids, thus maintaining the fluidity of membranes. The PNPLA3, TM6SF2 and MBOAT7 proteins are represented in green and their physiological functions are indicated by green arrows (**B**). The prolonged ethanol (Et-OH) exposure impairs insulin sensitivity, enhancing the flux of free fatty acids from adipose tissue and de novo lipogenesis, induced by sterol regulatory element-binding protein (*SREBP-1c*). These events lead to fat accumulation which may be exacerbated by the presence of genetic modifiers. The *PNPLA3* 148M variant increases hepatic TG content upon accumulation of the mutant protein on the surface of lipid droplets. Indeed, 148M proteins interfere with lipid remodeling in fatty-laden hepatocytes and inhibit the activity of other lipases, reducing TG turnover and dismissal. Moreover, the 148M variant impairs the amount of VLDL released, exacerbating fat deposition. The *TM6SF2* E167K variant impairs physiological VLDL secretion and affects cholesterol metabolism and TG synthesis. The intronic rs641738 variant in *MBOAT7* reduces membrane fluidity and dynamism by altering phospholipid acyl-chains remodeling and enhancing the amount of free arachidonic acid (AA), triggering hepatic inflammation. The PNPLA3, TM6SF2 and MBOAT7 mutated proteins are coloured in red and their aberrant effects are highlighted by red arrows. Dashed arrows indicate the bidirectional flux of molecules from extracellular to intracellular spaces and *viceversa*. The combined effect of *PNPLA3*, *TM6SF2* and *MBOAT7* genetic variants may predispose to a worsening risk of progressive liver damage and HCC (**B**). Modified by Dongiovanni, P., *Int. J. Mol. Sci.* **2017**.

Genome-wide association studies (GWAS) have identified genetic risk loci for ALD and NAFLD [54,55,56]. From these studies, the non-synonymous genetic variant I148M in the *Patatin-like phospholipase domain containing-3* (*PNPLA3*) gene has emerged as the major risk factor for chronic liver disease progression. Subsequently, a variant causing an amino acid change (E167K) in the *Transmembrane 6 superfamily member 2* (*TM6SF2*) gene, has been associated with the development and the severity of NAFLD. Lately, the rs641738 variant in the *Membrane bound O-acyltransferase domain containing 7-Transmembrane channel-like 4* (*MBOAT7/TMC4*) locus has been related to a higher risk of cirrhosis in alcohol abusers and with liver disease progression in NAFLD [57,58]. The impact of these genetic variants on fat accumulation is schematically represented in Figure 1.

### 4.1. PNPLA3

A GWAS of non-synonymous single nucleotide polymorphisms (SNPs) in NAFLD identified the rs738409 variant (C.444 C>G p.Ile148Met) in the *PNPLA3* gene as the major genetic determinant of inter-individual and ethnicity-related differences in hepatic fat content and the major predictor of predisposition to progressive liver damage, influencing also the response to therapeutic approaches [54]. Steatosis was assessed using proton magnetic resonance spectroscopy in a cohort of 2111 patients in the Dallas Heart Study. *PNPLA3* rs738409 was significantly associated with increased hepatic triglyceride content and liver inflammation. The I148M variant has been subsequently associated with steatosis, nonalcoholic steatohepatitis (NASH), fibrosis, and HCC also in various candidate gene studies [59,60,61]. This variant is most common in Hispanics and less frequent in Caucasians and African Americans pondering what is the frequency of steatosis in these ethnic groups. These data could partially explain the inter-ethnic variability in NAFLD severity and provide an enlightenment for the differences observed even in ALD.

The rs738409 variant in *PNPLA3* gene has been repeatedly associated with NAFLD development and progression but not with insulin resistance and the MetS components. It is widely recognized that NAFLD is a multisystem condition with extra-hepatic complications. Di Costanzo et al. demonstrated that patients with NAFLD and *PNPLA3* CC genotype had greater carotid intima-media thickness compared to blood donors with *PNPLA3* GG genotype [62]. It could be speculated that the MetS-related NAFLD and the *PNPLA3*-related NAFLD may differently impact on cardio-metabolic risk and that NAFLD is more associated to atherosclerosis when it is linked to MetS traits independently of *PNPLA3* genotype [63]. 

Several candidate gene studies have identified *PNPLA3* as a modifier of ALD progression. Firstly, Tian et al. found that the rs738409 variant in *PNPLA3* was associated with ALD and alcohol-related cirrhosis (OR = 2.25; *p* = 1.7 × 10^−10^) in European and Native American ancestry individuals with a history of alcoholism and the association remained significant after adjusting for ethnicity [64]. In European Caucasians, the *PNPLA3* rs738409 variant was associated with alcoholic liver cirrhosis and elevated aminotransferase levels [65]. Falleti et al. found that in Italian cirrhotic patients the G allele was over-represented in alcoholic/metabolic versus viral liver diseases. In addition, the rs738409 variant was identified as an independent predictor of HCC occurrence pointing out for the first time a role of the I148M *PNPLA3* variant in the risk of HCC (OR = 1.76; *p* < 0.05) also in non-drinking alcoholic cirrhosis [12,66,67]. The relationship between *PNPLA3* rs738409 and progressive ALD has been further confirmed in a European GWAS, with independent validation [58]. In patients with established alcohol-related cirrhosis, the rs738409 variant in *PNPLA3* was associated with an increased risk to develop HCC [68,69,70]. A meta-analysis of five studies confirmed this association which was more pronounced in ALD (OR = 2.2; [95% CI: 1.8–2.67]; *p* = 4.71 × 10^−15^) than in chronic hepatitis C patients (OR = 1.55; [95% CI: 1.03–2.34]; *p* = 3.52 × 10^−2^) [71]. Management guidelines recommend implementation of ultrasound screening to early detect HCC and genotyping for the rs738409 PNPLA3 variant will allow for more precise HCC risk-stratification of patients, in particular those with alcohol-related cirrhosis [66,72,73]. 

The *PNPLA3* gene is located on chromosome 22 and encodes a 481-amino acid protein, also called adiponutrin, which has hydrolase activity. In particular, *PNPLA3* is an intracellular membrane lipase, localized in the ER and at the surface of lipid droplets, implicated in lipid remodeling in hepatocytes and in adipocytes. The molecular mechanism underlying the association between the *PNPLA3* I148M variant and progressive liver disease is still undefined. While the I148M variant determines a loss of enzymatic activity [74] and steatosis development seems to depend upon the accumulation of the mutant 148M *PNPLA3* on the surface of lipid droplets [75], the deletion of the protein has no pathological phenotype. Indeed, I148M *PNPLA3* proteins interfere with lipid remodeling in fatty-laden hepatocytes and inhibit the activity of other lipases, reducing triglyceride turnover and dismissal. The accumulation of the I148M mutated protein seems to be due to the less accessibility to ubiquitin ligases and to impaired proteasomal degradation [75]. In addition, human studies on the rs738409 variant have shown that *PNPLA3* has a role in the regulation of very low-density lipoprotein (VLDL) secretion. Indeed, the 148M variant impairs the amount of VLDL released, leading to steatosis development [76].

*PNPLA3* is strongly expressed and synthesized even in primary HSCs, mainly involved in fibrogenesis, and catalyzes the hydrolysis of retinyl esters, regulating retinol release. The I148M could hamper the dismissal of retinol and lipids from intracellular lipid droplets, resulting in a more pronounced fibrogenic phenotype [77].

### 4.2. TM6SF2

Exome-wide association studies identified the rs58542926 (c.499C>T) genetic variant in the *TM6SF2* gene, which encodes the aminoacidic substitution E167K (p.Glu167Lys), as a determinant of hepatic triglyceride content, elevated serum aminotransferases, and lower serum lipoproteins [55]. *Tm6sf2* silencing in mice increased hepatic fat accumulation by three-fold and decreased VLDL secretion by 50% suggesting that *TM6SF2* is involved in VLDL release and that impaired *TM6SF2* function may lead to NAFLD development. The positive association between the rs58542926 variant and the amount of hepatic triglyceride content remained significant even after adjustment for the *PNPLA3* rs738409 genotype, indicating that the E167K variant’s effect on steatosis onset is independent of *PNPLA3*. Additionally, carriers of the rs58542926 showed lower circulating lipids and a reduced cardiovascular risk [78]. 

CRISPR-Cas9 generated *Tm6sf2* knock-out mice displayed reduced plasma levels of total cholesterol. In addition, the expression of genes involved in cholesterol metabolism was altered in these mice, leading to the hypothesis that *TM6SF2* may play a role in this process [79].

Similarly to *PNPLA3,* candidate gene studies confirmed the association between the rs58542926 *TM6SF2* variant and hepatic fat content, also in the pediatric population [80,81,82]. Furthermore, carriers of the E167K variant also displayed an increment of risk of NASH, more advanced fibrosis and cirrhosis [83,84].

Following on from the identification of *TM6SF2* as a modifier of NAFLD, a recent study has established that the rs58542926 variant was independently associated with HCC in patients with ALD (OR = 1.79 [CI 95%:1.25–2.56]) [69]. Finally, a GWAS identified the E167K variant as a genetic locus associated with increased risk of alcohol-related cirrhosis in individuals of European descent [58]. The combined effect of *PNPLA3* and *TM6SF2* genetic variants may predispose to a worsening risk to develop HCC in alcoholic cirrhosis [85]. In a very recent paper, Stickel et al. found that the development of HCC was independently associated with rs738409 PNPLA3 (OR = 1.84 [CI 95%: 1.55–2.18]) and rs58542926 TM6SF2 (OR = 1.66 [CI 95%: 1.30–2.13]) variants. Patients with alcohol-related cirrhosis who carry the rs58542926 TM6SF2 variant have an additional risk factor for the development of HCC and carriage of both PNPLA3 rs738409 and TM6SF2 rs58542926 accounts for half of the attributable risk for HCC in this population [72].

### 4.3. MBOAT7

A GWAS for alcohol-related cirrhosis in European descent (712 cases and 1,426 controls) identified the rs641738 C>T SNP in the *MBOAT7/TMC4* locus as a new genetic risk factor which predisposes alcohol abusers to cirrhosis [58]. This association remained significant in a subset of patients, irrespectively of gender, age, type 2 diabetes, excess in body weight and socioeconomic factors, consistent with a key role of heritability. The rs641738 variant in the *MBOAT7-TMC4 locus* has been subsequently correlated to the increased risk of the entire spectrum of NAFLD, without influencing cardiovascular risk [57,86]. Both mRNA expression and protein synthesis were reduced in carriers of T risk allele suggesting that the mechanism underlying the association with hepatic fat content and liver damage progression is related to lower enzymatic activity due to hampered protein levels. The rs641738 variant is located in the 19q13.42 region and contains *TMC4* and *MBOAT7*. *MBOAT7* also referred to as *Lysophosphatidylinositol Acyl-Transferase 1* (*LPIAT1*), is an enzyme involved in the re-acylation of phospholipids as part of the phospholipid acyl-chains remodeling pathway, known as the Lands’ cycle [87], which has a high specificity for arachidonoyl-CoA as an acyl donor. It could be hypothesized that the rs641738 variant leads to a reduced *MBOAT7* expression and predisposes to NAFLD/NASH by affecting the acyl remodeling of phosphatidylinositols in the liver, favoring the increase in free arachidonic acid, a potent driver of hepatic inflammation [57,88]. Finally, it has been described that the *MBOAT7* rs641738 T allele conferred an approximately 80% increased risk of HCC in Italian NAFLD patients and was associated with HCC development in non-cirrhotic patients affected by ALD. The effect of this variant on HCC risk disappears with disease progression and it has no effect on the late stages of fibrosis [89]. 

*PNPLA3*, *TM6SF2*, and *MBOAT7* are genetic modifier of both ALD and NAFLD which share the same histological pattern. These genes are involved in lipid remodeling suggesting that an aberrant hepatic lipid handling exerts a crucial role in ALD and NAFLD pathogenesis. Further studies are required to understand the functional role of these variants in order to identify new therapeutic targets and take a step forward towards personalized medicine.

### 4.4. Other Genetic Factors Involved in Alcohol-Related Liver Injury

Several case-control studies have been performed in order to investigate the possible association between alcohol-induced liver injury and candidate gene variants involved in alcohol metabolism, ethanol-induced oxidative stress, inflammation, and fibrosis [90,91]. However, many findings have not been replicated because of inadequate sample size and statistical power or inappropriate selection of control groups.

Variants in alcohol-degrading enzymes, such as cytosolic *ADH* and mitochondrial *ALDH*, were associated with alcoholism and alcohol-related HCC in heavy drinkers but not with ALD [92,93,94].

The association between ALD and polymorphisms in those genes mediating cellular antioxidant defenses, such as *Manganese-dependent Superoxide Dismutase* (MnSOD encoded by *SOD2* gene) and members of *Glutathione S-transferases* family (*GSTs),* has not been well-established. Indeed, the common nonsynonymous variant (rs4880) in *SOD2* gene, resulting in the Ala16Val (C47T) aminoacidic substitution, was previously associated with progressive ALD in a small case-control study [95]. However, it failed to be confirmed in a large cohort study, comparing 281 patients affected by ALD with advanced fibrosis/cirrhosis to 218 binge drinkers without evidence of ALD [96]. 

The presence of the A risk allele at -627 position (C>A) in the promoter of *Interleukin-10* (*IL10*) was closely related to lower IL10 production and favored the pro-inflammatory process by driving HSCs activation and fibrosis. Indeed, carriers of the minor (A) allele showed a more severe liver disease [97,98,99]. Also, the association between the G>A substitution at position -238A in the *TNFα* promoter and ASH has been independently validated [100,101].

Despite progressive fibrosis represents one of the main hallmarks of ALD, few case-control studies focused on candidate genes involved in wound-healing and matrix remodeling processes such as TGFβ and *Matrix Metalloproteinase-3* (*MMP-3*). However, Arg25Pro (c.915 G>C) aminoacidic substitution in TGFβ1 and the rs35068180 (−1171 5A/6A) polymorphism in the promoter of *MMP-3* was not associated with ALD [102,103]. 

Finally, the rs2228603 (c.274 C>T, p.Pro92Ser) in Neurocan (NCAN) gene, commonly associated with NAFLD, has further been identified to represent an independent risk factor for HCC onset even in patients affected by alcohol-related cirrhosis (OR = 1.840; 95% CI = 1.220–2.777; p = 0.004), displaying a 15.1% of frequency in ALD-HCC subjects [104]. Genetic five-mapping showed that NCAN rs2228603 lies within 50 kb and is in strong linkage disequilibrium with rs58542926 TM6SF2 (D′=0.926, r2=0.798) [83] and it is actually the TM6SF2 signal that pops up with NCAN [105]. 

## 5. Epigenetic Factors Involved in ALD Pathogenesis

Epigenetic mechanisms which are deregulated by alcohol in the liver may contribute to ALD pathogenesis and progression. These mechanisms have been identified in parenchymal and non-parenchymal cells in the liver and contribute to steatosis, inflammation, and oxidative stress. Epigenetic modifications are hereditable changes that impact on gene expression without altering nucleotides sequence. Examples include DNA methylation, histone modifications and RNA silencing by microRNAs (miRNAs). Here we focus our attention on miRNAs as epigenetic modulators in ALD.

**Figure 2:**
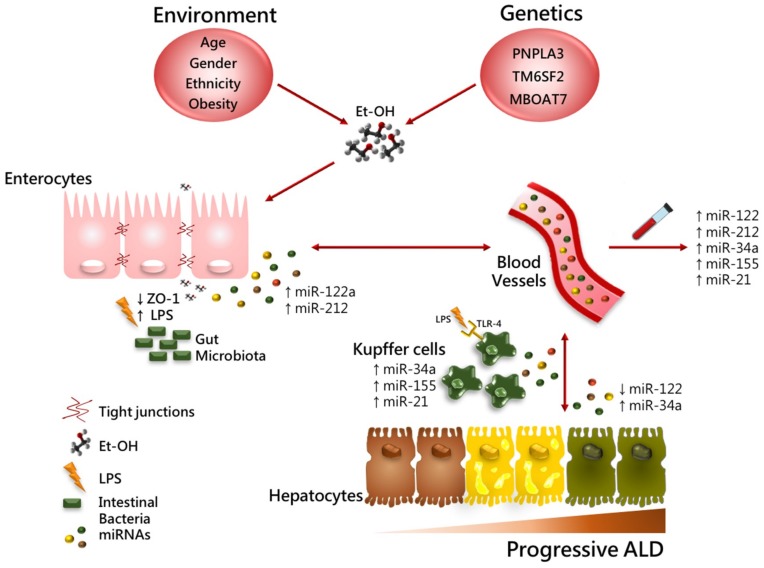
Role of miRNAs in progressive ALD. Environmental, genetic, and epigenetic factors along with excessive alcohol abuse contribute to ALD pathogenesis and progression. In this figure, we reported a schematic illustration of candidate miRNAs demonstrated to affect Et-OH-induced liver injury in both different hepatic and small intestinal cell types: hepatocytes, inflammatory cells (i.e., Kupffer cells), and enterocytes. Several miRNAs can be secreted into the circulation through exosomes and microvesicles triggering steatosis onset, inflammation, fibrosis, and carcinogenesis. Increasing intestinal expression of miRNAs (miR-122 and miR-212) affect gut barrier integrity by loosening the tight junctions of Zonula Occludens 1 (ZO-1) and enhanced Lipopolysaccharides (LPS) release into the circulation. LPS induced Toll-like Receptor 4 (TLR4) activation in hepatic Kupffer cells and macrophages. miRNAs even support hepatic inflammation via NF-kB; IL6/STAT3 signaling and oxidative stress (i.e., TNF-α and ROS production) and can contribute to the worsening of hepatic injury by a strict but firm regulation of cell–cell cross-talk. Circulating miRNAs can be easily detected in blood circulation as diagnostic, prognostic, and predictive biomarkers.

miRNAs are short non-protein coding, single-strands RNAs 19–22 nucleotides long, that regulate gene expression [106]. miRNAs function via base-pairing with complementary sequence within mRNA molecules, thereby silencing them through transcript destabilization or less efficient translation of the mRNA into proteins [106,107,108]. miRNAs’ dysregulation has been shown to have high prognostic and predictive value in a broad spectrum of liver diseases, including viral hepatitis, ALD and NAFLD, fibrosis, and HCC [109]. miRNAs are also stable in the circulation (blood, urine) thus representing potential biomarkers for ALD [110,111]. Several studies have shown that miRNAs expression could be altered by alcohol consumption [112,113,114]. Up to now, miRNAs mainly related to ALD include miR-155, miR-34a, miR-122, miR-212, and miR-21 (Table 1 and Figure 2). Of note, these miRNAs have also been implicated in NAFLD reinforcing a common shared mechanism between these two diseases and the pleiotropic effect of several miRNAs.

### 5.1. miR-155

It has been largely demonstrated that miR-155 plays an important role in ALD [139]. miR-155 expression was increased in hepatocytes and Kupffer cells in both ethanol-fed mice and RAW 264.5 macrophages treated with alcohol [112,115]. The increased intestinal permeability due to alcohol consumption leads to higher LPS which bind to *Toll-like receptor4* (*TLR4*) and upregulate miR-155 through *Nuclear factor kappa-light-chain-enhancer of activated B cells* (*NF-κB*) thus promoting the release of TNFα, ROS and oxidative stress production and the activation of Kupffer cells and HSCs [115,116]. Bala et al. [117] showed that miR-155 knock-out mice presented reduced alcohol-induced steatosis, liver injury, and oxidative stress compared to the control group. This study demonstrated that chronic ethanol-enriched diet increased the number of inflammatory monocytes, macrophages and neutrophils in the liver of wild type mice whereas no increase was revealed in miR-155 knock-out mice. The effects caused by miR-155 in hepatocytes and inflammatory cells were probably related to the PPAR signaling pathway. *PPARα* and *PPARγ,* which are involved in the processes of fat metabolism, oxidative stress, inflammation, and fibrosis, are targets of miR-155 [117]. In hepatocytes, miR-155 upregulation leads to a reduced expression of *PPARα* and to induction of genes implicated in lipid metabolism and uptake, such as *Fatty acid binding protein 4* (*FABP4), Acetyl-CoA carboxylase* 1 *(ACC1)*, and *Low-density lipoprotein receptor (LDLR)*. In macrophages, the upregulation of miR-155 induced by alcohol exposure dampened the expression of *PPARγ* and other fibrosis-related genes. Further studies demonstrated that chronic alcohol feeding enhanced mRNA levels of *Tgfβ* in wild-type mice, whereas this increase was either prevented in miR-155 knock-out mice [117]. Although there have been no reports on miRNA-driven therapy for the management of ALD, it has been shown that the inhibition of miR-155 in Kupffer cells and macrophages upon ethanol exposure ameliorates LPS-induced *TNFα* production [115]. The upregulation of miR-155 in macrophages has been often flanked by hampered miR-125b levels in response to endotoxic *stimuli* [131]. Likewise, higher levels of hepatic miR-181a has been shown to alter glucose and lipid homeostasis and to increase LPS-induced TLR4/NF-kB activation by disrupting intestinal barrier [135,140].

### 5.2. miR-34a 

miR-34a is a member of the miR-34 family and represents a key regulator of tumor growth inhibition and p53-mediated apoptosis [141]. Upregulation of miR-34a was shown in alcohol-induced liver injury and played an important and complex role in ALD [118,120]. One of the target genes of miR-34a is *Sirtuin 1* (*SIRT1*), encoding the Sirtuin protein [121]. Reduced levels of *SIRT1* have been reported in patients with ALD and hepatic depletion of *Sirt1* in mice promotes steatosis, inflammation, and fibrosis in response to ethanol challenge [119]. It has been described that miR-34a can be methylated, as its 5′-promoter region is embedded in a CpG island. Hypomethylation of miR-34a induced by ethanol exposure was associated with its higher expression [118]. Finally, under metabolic stress conditions, the miR-34a/*Hepatocyte nuclear factor 4 alpha* (*HFN4α*) pathway is activated supposedly exerting a role in lipoprotein’s metabolism and in the pathogenesis of NAFLD. However, miR-34a involvement in ALD onset is still undefined [142].

### 5.3. miR-122-miR-212

miR-122 represents 70% of all miRNAs in mature hepatocytes. Mice with liver specific deletion of miR-122 develop steatosis at birth which spontaneously progress to fibrosis and HCC. miR-122 expression was dampened in patients with alcohol-related cirrhosis and in hepatocytes isolated from ethanol-fed mice [122], in which it regulates both synthesis and export of lipids and cholesterol homeostasis [114]. Overexpression of miR-122 attenuated steatosis, ALT levels and key inflammatory cytokines as monocyte chemoattractant protein 1 (MCP1) and IL-1β in ethanol-fed mice [122]. In a mouse model of alcohol-induced liver injury Ambade et al. found that miR-122 downregulation is enable to induce the expression of *Hypoxia-inducible factor 1 alpha* (*Hif1α*), which contributes to the acceleration of hepatobiliary cancer [123]. Alcohol consumption increases gut permeability and translocation of bacterial products as LPS into the intestinal and portal circulation. miR-122a can also affect intestinal permeability by targeting occludins, transmembrane tight junction proteins, as shown in both Caco-2 cells and mice enterocytes [112]. Probiotics can be exploited to regulate intestinal permeability by downregulating miR-122a, thus promoting the expression of occludins and preventing the development of ALD [124]. As a consequence of alcohol feeding, intestinal rising levels of miR-212 were reported to target tight junction proteins like Zonula Occludens-1 (ZO-1). Therefore, patients with ALD displayed an altered gut microbiota composition and enhanced intestinal expression of miR-212 and decreased ZO-1 protein levels [143]. This study suggests that elevated concentrations of miR-212 in the small intestine and colon may influence tight junction proteins and negatively affect intestinal barrier integrity in ALD [125]. Since Miravirsen, a miR-122 inhibitor used for the treatment of chronic hepatitis C, has shown promising results, it has been proposed as a potential therapeutic strategy for other chronic liver diseases, including ALD. Indeed, Miravirsen might exert its therapeutic effects by interrupting the cross-talk between hepatocytes, Kupffer cells, and HSCs that could induce the worsening of liver damage [144].

### 5.4. miR-21

miR-21 is one of the most frequently upregulated miRNA in solid tumors, including HCC. The human miR-21 gene is located on chromosome 17, close to p53. The regulation of miR-21 by p53 suggests that it might play a role in the modulation of hepatic cell survival and regeneration during liver injury [126]. Several studies revealed that prolonged alcohol feeding enhanced miR-21 levels in the liver [127,128]. Similarly, the treatment of human hepatocytes and HSCs with ethanol and IL6 significantly upregulated miR-21. Overexpression of miR-21 decreased ethanol-induced apoptosis in both cell types and the expression of miR-21 was significantly enhanced upon *signal transducer and activator of transcription 3* (*STAT3*) activation in these cells. Such findings suggest that the mechanism of miR-21 induction with alcohol exposure in the liver involves the activation of IL6/STAT3 signaling pathway. Additionally, miR-21 was shown to target the 3′ UTRs of *FAS ligand G* (*FASLG*) and *death receptor 5* (*DR5*) whose expression was downregulated by ethanol. miR-21 is induced with ethanol exposure *via* IL6/STAT3 signaling and targets extrinsic apoptotic mediators like FASLG and DR5 in order to prevent hepatic cells apoptosis and to favor liver regeneration in ALD [128].

### 5.5. Other miRNAs Implicated in Alcohol-Induced Liver Injury

Several other miRNAs are modulated by alcohol consumption and their activation/inhibition could represent a novel therapeutic strategy for the management of alcohol-induced liver injury. Indeed, while the expression of miR-181, miR-217, and miR-223 was found to be elevated, miR-29a/c, miR-125, miR-126, miR-199a, miR-200a, and miR-375 were decreased in both humans and rodents after ethanol exposure. In particular, members of miR-29 family are closely related to *TGFβ1*/*SMAD3* signaling and their anti-inflammatory and anti-fibrotic effects are critically dampened by *TGFβ1* during fibrogenesis in several tissues. Thus, their upregulations may represent a possible therapeutic approach [129]. Consistently, miR-126 was found to be downregulated in fibrogenic processes and it may be considered a marker of HSCs activation, proliferation and contractility [134].

miR-223 is one of the most abundant miRNA in neutrophils and modulate ethanol-induced peripheral neutrophils activation and their infiltration into the liver, targeting NF-kB and the nucleotide-binding oligomerization domain-like (NOD) receptor protein 3 inflammasome. Specifically, it regulates macrophages polarization and inflammasome activation and it was found to be elevated in serum and in neutrophils of both alcoholics and ethanol-fed mice. However, human neutrophil expression of miR-223 remains still controversial and it could be the result of different exposure time to alcohol [111]. miR-223 deficient mice display a more pronounced hepatic neutrophil infiltration and more severe liver damage by favoring IL6 and phagocytic oxidase (phox) p47phox expressions and oxidative stress [137]. Since the specific protective mechanism of miR-223 in alcohol-induced liver injury remains still controversial, further investigations will be crucial to identify strategies for the treatment of ALD. On the contrary, it has been demonstrated that miR-223 is downregulated in HCC versus adjacent tissues and its overexpression in hepatoma cell lines increases the sensitivity to anticancer drugs [138].

Several studies have elucidated that HCC derived from different etiologies possesses a characteristic miRNA fingerprint. For example, downregulation of miR-199 was determined to be related to advanced liver diseases and HCC, to mediate cell cycle arrest and apoptosis and consequently influencing patients’ prognosis [112,130]. Moreover, Ladeiro and collaborators highlighted that miR-126 and miR-375 deregulations were linked to alcohol-related HCC and beta-catenin gene mutations, respectively [133]. 

## 6. Conclusions

Genetics and environmental factors are strongly entangled in ALD pathogenesis and progression. Epigenetics changes interact with genetic modifiers thus increasing individual susceptibility to ALD and possibly explaining the phenotypic variability. Currently, the treatment of ALD is based on alcohol abstinence, pharmacological treatment (corticosteroids, prednisolone, pentoxifylline), or liver transplantation in the presence of severe and advanced liver disease, respectively [145,146,147,148]. Moreover, medical and surgical treatments have more efficacy in total alcohol abstinence [149]. Therefore, there is an urgent need to identify non-invasive biomarkers to early diagnose and treat individuals at higher risk of ALD. The genetic data collected in the last years have shed light on the inherited aspects of ALD. Nonetheless, miRNAs have become appealing molecules candidates for diagnosis and staging of liver diseases and their combination with the genetic profile will promote the development of novel personalized therapeutic approaches. However, there are no clinical trials which have addressed miRNAs as therapeutic targets in ALD and further studies are required before clinical application.

## Figures and Tables

**Table 1:** miRNAs regulated by alcohol consumption in ALD experimental models

miRNAs	Epigenetic Mechanism	Function	Experimental Model	ALD Severity	Ref.
miR-155	Upregulated	Induced *TNFα* and *PPARα* and hampered *PPARγ* expression	Hepatocytes, macrophages, Et-OH fed mice	Steatosis, inflammation, oxidative stress, fibrosis, cirrhosis	[112,115,116,117]
miR-34a	Upregulated	Reduced *SIRT1* gene	Et-OH fed mice and rats	Steatosis, NASH, Fibrosis	[118,119,120,121]
miR-122	Downregulated	Induced genes involved in lipid metabolism, cholesterol homeostasis, cytokines and *HIF1α*. Reduced tight junction proteins	ALD patients, Et-OH fed mice	Steatosis, inflammation, altered gut permeability, fibrosis, cirrhosis	[112,114,122,123,124]
miR-212	Upregulated	Dampened tight junction proteins	ALD patients	Altered gut permeability	[125]
miR-21	Upregulated	Modulation of cell proliferation, apoptosis and survival	Hepatocytes and HSCs treated with alcohol Et-OH fed rats and mice,	Liver Injury and HCC	[126,127,128]
miR-29	Downregulated	Modulation of hepatic inflammation and HSCs activation	ALD patients, CCl_4_-induced fibrosis in rodents	Liver Injury, altered gut permeability fibrosis	[129]
miR-199a	Downregulated	Regulation of cell cycle and apoptosis through *HIF1α* induction	ALD patients, HCC cell lines and AML-12, Et-OH fed mice	Inflammation, cirrhosis, HCC	[112,130]
miR-125b	Downregulated	Increased *TNFα* production	Et-OH fed mice, macrophages	Liver Injury, HCC	[131,132]
miR-126	Downregulated	Activation of HSCs and induction of Et-OH related HCC	ALD patients, HSCs, CCl_4_-induced fibrosis in rodents	Fibrosis, HCC	[133,134]
miR-181a	Upregulated	Increased LPS sensitivity via TLR4/NF-kB	Et-OH fed mice	Steatosis, altered gut permeability, Inflammation	[135]
miR-200a	Downregulated	Induction of hepatocytic apoptosis	ALD patients, Et-OH fed mice	HCC	[113,133]
miR-217	Upregulated	Inhibition of *SIRT1*	Hepatocytes, macrophages, Et-OH fed mice	Steatosis, Inflammation	[136]
miR-375	Downregulated	Regulation of *HIF1α* and association with beta-catenin genes mutations	ALD patients	Inflammation, HCC	[133]
miR-223	Upregulated	Hepatocytic apoptosis, neutrophils hepatic infiltration	ALD patients, Et-OH fed mice, CCl_4_-induced acute liver injury	Liver injury, inflammation, HCC development	[137,138]

## Abbreviations

ACC1	Acetyl-CoA Carboxylase Alpha
ADH	Alcohol Dehydrogenase
ALD	Alcoholic Liver Disease
ALDH	Aldehyde Dehydrogenase
ASH	Alcoholic Steatohepatitis
AUD	Alcohol Use Disorder
BMI	Body Mass Index
CYP2E1	Cytochrome P450 2E1
DR5	Death receptor 5
ER	Endoplasmic reticulum
Et-OH	Ethanol
FABP4	Fatty Acid Binding Protein 4
FASLG	Fas Cell Surface Death Receptor Ligand G
FFAs	FFAs Free Fatty Acids
GST	Glutathione- S-Transferases
GWAS	Genome-Wide Association Studies
HCC	Hepatocellular Carcinoma
HIF-1α	Hypoxia Inducible Factor 1 Subunit Alpha
HNF4α	Hepatocyte Nuclear Factor 4 Alpha
HSCs	Hepatic Stellate Cells
IL1β	Interleukin-1 Beta
IL6/STAT3IL10	Interleukin-6/ Signal Transducer and Activator Of Transcription 3Interleukin-10
LDLR	Low Density Lipoprotein Receptor
LPIAT1	Lysophosphatidylinositol acyl- transferase 1
LPS	Lipopolysaccharides
MBOAT7/TMC4	Membrane bound O-acyltransferase domain containing 7-Transmembrane channel-like 4
MCP1	Monocyte chemoattractant protein 1
MetS	Metabolic Syndrome
miRNAs	microRNAs
MMP-3	Matrix Metalloproteinase-3
MnSOD	Manganese-Dependent Superoxide Dismutase
NAFLD	Nonalcoholic Fatty Liver Disease
NASH	Nonalcoholic Steatohepatitis
NCAN	Neurocan
NF-κB	Nuclear Factor Kappa-Light-Chain-Enhancer Of Activated B Cells
NOD	Nucleotide-binding Oligomerization Domain-like
OR [CI 95%]	Odds Ratio [Confidence Interval 95%]
p47phox	Phagocytic oxidase p47
PNPLA3	Patatin-like phospholipase domain containing-3
PPARα	Peroxisome Proliferator Activated Receptor-alpha
PPARγ	Peroxisome Proliferator Activated Receptor-gamma
ROS	Reactive Oxygen Species
SIRT1	Sirtuin 1
SNP	Single Nucleotide Polymorphism
SOD2	Superoxide Dismutase 2
SREBP-1c	Sterol regulatory element-binding protein
TG	Triglycerides
TGFβ	Transforming Growth Factor-Beta
TLR4	Toll-like receptor4
TM6SF2	Transmembrane 6 superfamily member 2
TNFα	Tumor Necrosis Factor alpha
UTR	Untranslated region
VLDL	Very Low-Density Lipoprotein
WHO	World Health Organization
ZO-1	Zonula Occludens-1
